# *Klebsiella pneumoniae* infection of murine neutrophils impairs their efferocytic clearance by modulating cell death machinery

**DOI:** 10.1371/journal.ppat.1007338

**Published:** 2018-10-01

**Authors:** Christopher N. Jondle, Kuldeep Gupta, Bibhuti B. Mishra, Jyotika Sharma

**Affiliations:** Department of Basic Biomedical Sciences, The University of North Dakota School of Medicine and Health Sciences, Grand Forks, North Dakota, United States of America; University of Toronto, CANADA

## Abstract

Neutrophils are the first infiltrating cell type essential for combating pneumoseptic infections by bacterial pathogens including *Klebsiella pneumoniae* (KPn). Following an infection or injury, removal of apoptotic infiltrates via a highly regulated process called efferocytosis is required for restoration of homeostasis, but little is known regarding the effect of bacterial infection on this process. Here we demonstrate that KPn infection impedes the efferocytic uptake of neutrophils in-vitro and in-vivo in lungs by macrophages. This impaired efferocytosis of infected neutrophils coincides with drastic reduction in the neutrophil surface exposure of apoptosis signature phospholipid phosphatidyserine (PS); and increased activity of phospholipid transporter flippases, which maintain PS in the inner leaflet of plasma membrane. Concomitantly, pharmacological inhibition of flippase activity enhanced PS externalization and restored the efferocytosis of KPn infected neutrophils. We further show that KPn infection interferes with apoptosis activation and instead activates non-apoptotic programmed cell death via activation of necroptosis machinery in neutrophils. Accordingly, pharmacological inhibition of necroptosis by RIPK1 and RIPK3 inhibitors restored the efferocytic uptake of KPn infected neutrophils in-vitro. Importantly, treatment of KPn infected mice with necroptosis inhibitor improved the disease outcome in-vivo in preclinical mouse model of KPn pneumonia. To our knowledge, this is the first report of neutrophil efferocytosis impairment by KPn via modulation of cell death pathway, which may provide novel targets for therapeutic intervention of this infection.

## Author summary

Inflammatory diseases caused by infectious or sterile injuries are often characterized by pathological accumulation of dead or dying infiltrating cells. Pneumonic sepsis caused by *Klebsiella pneumoniae* (KPn), an opportunistic pathogen, has similar etiology, however, the underlying mechanism remains unknown. Here we report that KPn infection subverts a protective host process termed efferocytosis, by which the phagocytic cells engulf and clear dead/dying cells thereby resolving inflammation and infection. Our results show that KPn infected neutrophils are cleared less efficiently via efferocytosis than the uninfected neutrophils. Mechanistic studies implicated a reduced exposure of “eat me” signal phosphatidyleserine (PS) via increased flippase activity and skewing of cell death pathway toward necroptosis in impaired efferocytosis of infected neutrophils. Accordingly, pharmacological reversal of PS exposure by flippase inhibition, treatment with necroptosis inhibitors restored the efferocytic clearance of KPn infected neutrophils, and improved the disease outcome in a preclinical model of pneumonic sepsis. To our knowledge this is the first report of KPn subversion of efferocytic clearance of neutrophils by impairing pro-efferocytic apoptotic signatures and activation of necroptosis machinery. This could lead to novel therapeutic targets against KPn infection and associated inflammation in pneumonic sepsis.

## Introduction

Pneumonia is the most frequent cause of sepsis [1–3], which is one of the oldest and most elusive syndromes and a major challenge in medicine [4]. With no effective therapies there are over 750,000 cases of sepsis each year in the United States alone, which accounts for 10% of all ICU patients, leading to a mortality rate between 20–50% depending on certain risk factors [5, 6]. In particular *Klebsiella pneumoniae* (KPn), an opportunistic pathogen, accounts for 5–20% of all Gram-negative sepsis cases [1, 3]. A notable emergence of antibiotic resistant strains of KPn in clinical settings has caused concerns over an already dwindling armamentarium of antibiotics. Thus, an understanding of host immune responses and pathogen-mediated manipulation thereof will likely provide novel therapeutic targets. In this regard, neutrophils are the first cell types to infiltrate the site of infection and contribute to the initial protective response. Indeed, in murine models of KPn infection, neutrophil-mediated responses are shown to be essential for initial control of the infection [7, 8]. We and others have shown that persistent accumulation of neutrophils and their over activation causes perpetuation of inflammation in pneumoseptic KPn infection [9, 10] [11–15]. Moreover, neutrophils have been reported to constitute a reservoir for this pathogen and aide in systemic dissemination of this infection [16]. This underscores the importance of neutrophil turnover in KPn pneumonia and sepsis.

Clearance of neutrophils by phagocytic cells, mainly macrophages, occurs via efferocytosis, which is a highly regulated receptor-dependent process [17, 18]. Aided by the action of phospholipid translocases such as flippases, apoptotic cells increase exoplasmic exposure of phosphatidylserine (PS), which is recognized as “eat-me” signal by macrophage cell surface receptors initiating their engulfment. The swift efferocytic clearance of infected and uninfected apoptotic cells prevents the release of pro-inflammatory mediators from dead cells as well as controls pathogens not destroyed through phagocytosis [19–21] [9, 22]. Apoptosis is thus considered an “immunologically silent” cell death mechanism [23]. Alternative cell death modalities, on the other hand, are typically characterized by rupture of outer membrane and release of intracellular contents recognized by the immune cells as danger signals eventuating in inflammatory responses [24]. Hence the immunological consequences of different cell death modalities differ, not only by virtue of their morphological features but also, by dictating the engagement of efferocytic cells. In that regard, modulating cell death modality may be a virulence mechanism of pathogens by which pathogens can subvert resolution of inflammatory response in their favor. Necroptosis is a programmed cell death which is mediated by receptor interacting protein kinase-3 (RIPK3) and its substrate mixed lineage kinase like (MLKL) [25]. Several studies have shown necroptosis in macrophages and epithelial cells to underlie inflammation and lung disease [26–32], however, activation of necroptosis in neutrophils and its implication in neutrophil turnover is not well understood and remains under active investigation. Moreover, while there is considerable research done on the efferocytosis pathways and its effect on sterile inflammatory disease development, studies on KPn subversion of this important protective host response are extremely limited. Lastly, whether KPn infection skews cell death to necroptosis in neutrophils and how it affects KPn pathogenesis and disease outcome is currently unknown.

In this study, we present key evidence to support several novel findings, which show that KPn infection impairs efferocytic uptake of neutrophils by inhibiting surface exposure of apoptosis signature PS exposure via modulation of flippase activity and activation of necroptosis pathway in neutrophils. With the use of pharmacological enhancers and blockers *in-vitro* and *in-vivo* in a preclinical mouse model of KPn pneumonia, our studies present evidence for a novel virulence mechanism of KPn which, by modulating programmed cell death, causes neutrophil turnover deficit. This defect can be targeted for therapies to treat KPn infection in the face of constant emergence of antibiotic resistant strains of this bacterium.

## Results

### KPn infection of neutrophils impairs their efferocytic uptake

**Fig 1:**
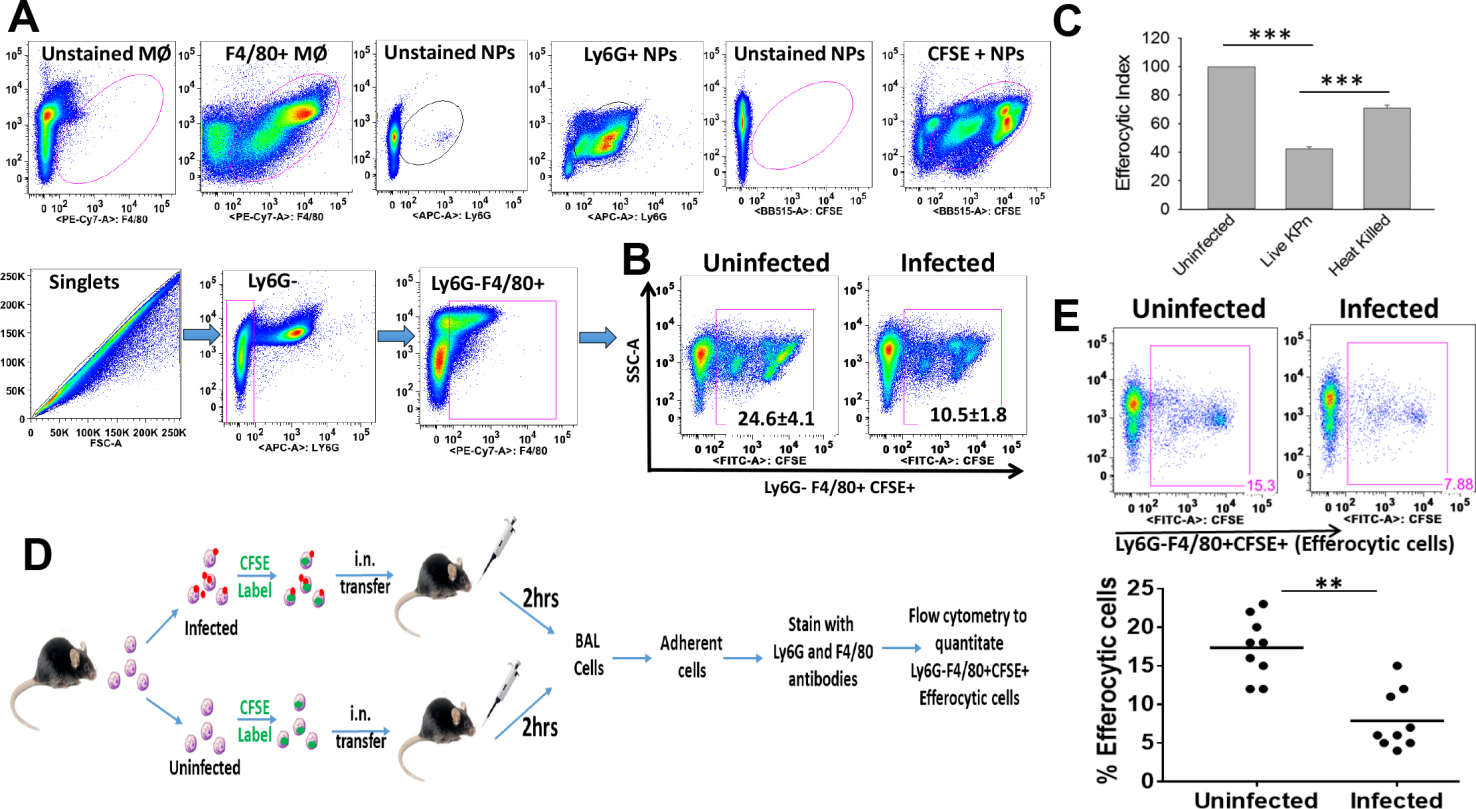
Live KPn infection impairs efferocytosis of neutrophils. **(A)** Schematic for gating F4/80+ macrophages that have internalized (hence are Ly6G-) CFSE labelled neutrophils (thus become CFSE+). Upper panel shows unstained and single stained cells. NP; Neutrophils, MØ, Macrophages. **(B)** Neutrophils left uninfected or infected with live or heat-killed KPn at MOI 10 for 3 hours, followed by labelling with the DNA dye CFSE and incubated with macrophages at 5:1 ratio for 2hrs. Macrophages were then processed for flow-cytometry by staining with anti-F4/80 and anti- Ly6G antibodies. Representative plot from one out of five independent experiments is shown. The numbers on pseudocolor plots show percent (average ± SEM) of Ly6G-F4/80+CFSE+ cells calculated from 5 independent experiments (3 technical replicates for each samples per experiment). The statistical analysis was done by Student’s *t* test (p<0.001). **(C)** Efferocytic Index shown as percent efferocytosis of infected neutrophils normalized to that of uninfected cells taken as 100%. Data shown is average ± SEM from 3–5 independent experiments. Statistics was done by ANOVA with Dunn’s post hoc analysis (***, p<0.001). **(D).** Schematic for in-vivo efferocytosis in the lungs of mice. CFSE labelled uninfected or KPn infected neutrophils were instilled intranasally in anaesthetized mice and lungs were lavaged 2 hrs later. Adherent cells were stained with F4/80 and Ly6G antibodies for flow cytometry. **(E)**. Representative flow cytometry dot plots showing percent of Ly6G-F4/80+CFSE+ efferocytic macrophages from mice instilled with uninfected or KPn infected neutrophils. The scatter plot shows percent efferocytic cells in each group of mice where each dot represents an animal. n = 9 from 3 independent experiments (**, p<0.005).

In order to determine the effect of KPn infection on efferocytosis of neutrophils, we compared the uptake of Carboxyfluorescein succinimidyl ester (CFSE) labelled uninfected or KPn infected neutrophils by macrophages using flow cytometry. Fig 1A (lower panel) shows the gating scheme using F4/80 and Ly6G antibodies to enumerate Ly6G-F4/80+CFSE+ macrophages that have internalized the CFSE-labelled neutrophils. Gating on singlets and Ly6G-negative cells allowed exclusion of adhered neutrophils since engulfed neutrophils will not be accessible to Ly6G antibody. As shown in Fig 1B, after 2 hrs of incubation with CFSE labelled neutrophils, 24.6% ± 4.1% macrophages stained positive for uptake of uninfected neutrophils. On the other hand, only 10.5% ± 1.8% macrophages had efferocytosed KPn infected neutrophils showing a 2.3 fold reduction of efferocytic uptake upon infection. This showed that infection of neutrophils with KPn caused a significant reduction in their efferocytic uptake by macrophages. Importantly, this infection-induced impairment was largely dependent on live bacteria and/or active bacterial replication as the neutrophils exposed to heat-killed KPn were efferocytosed more efficiently (efferocytic index of 70.9 ± 2.3, normalized to percentage uptake of uninfected neutrophils) as compared to those exposed to the live bacteria (efferocytic index 42.8 ± 1.4) (Fig 1C). Since bacterial factors likely involved in efferocytic impairment are not the focus of current study, we restricted further comparison between uninfected neutrophils and those infected with live bacteria only.

To investigate the physiological relevance of these findings we corroborated these data in a controlled *in-vivo* setting by intranasal administration of CFSE-labelled uninfected or KPn infected neutrophils in mouse lungs followed by flow cytometry analysis of alveolar macrophages (schematic shown in Fig 1D). Consistent with our *ex-vivo* results, significantly reduced numbers of Ly6G-F4/80+CFSE+ alveolar macrophages were recovered from the lungs of mice instilled with KPn infected neutrophils as compared to those that received uninfected neutrophils (Fig 1E). Together, these data strongly suggested that KPn infection significantly reduced the efferocytic clearance of neutrophils by macrophages in-vitro as well as *in-vivo*.

### Impaired efferocytosis of KPn infected neutrophils is linked to reduced externalization of PS *via* modulation of flippase activity and decreased activation of apoptotic caspases

**Fig 2:**
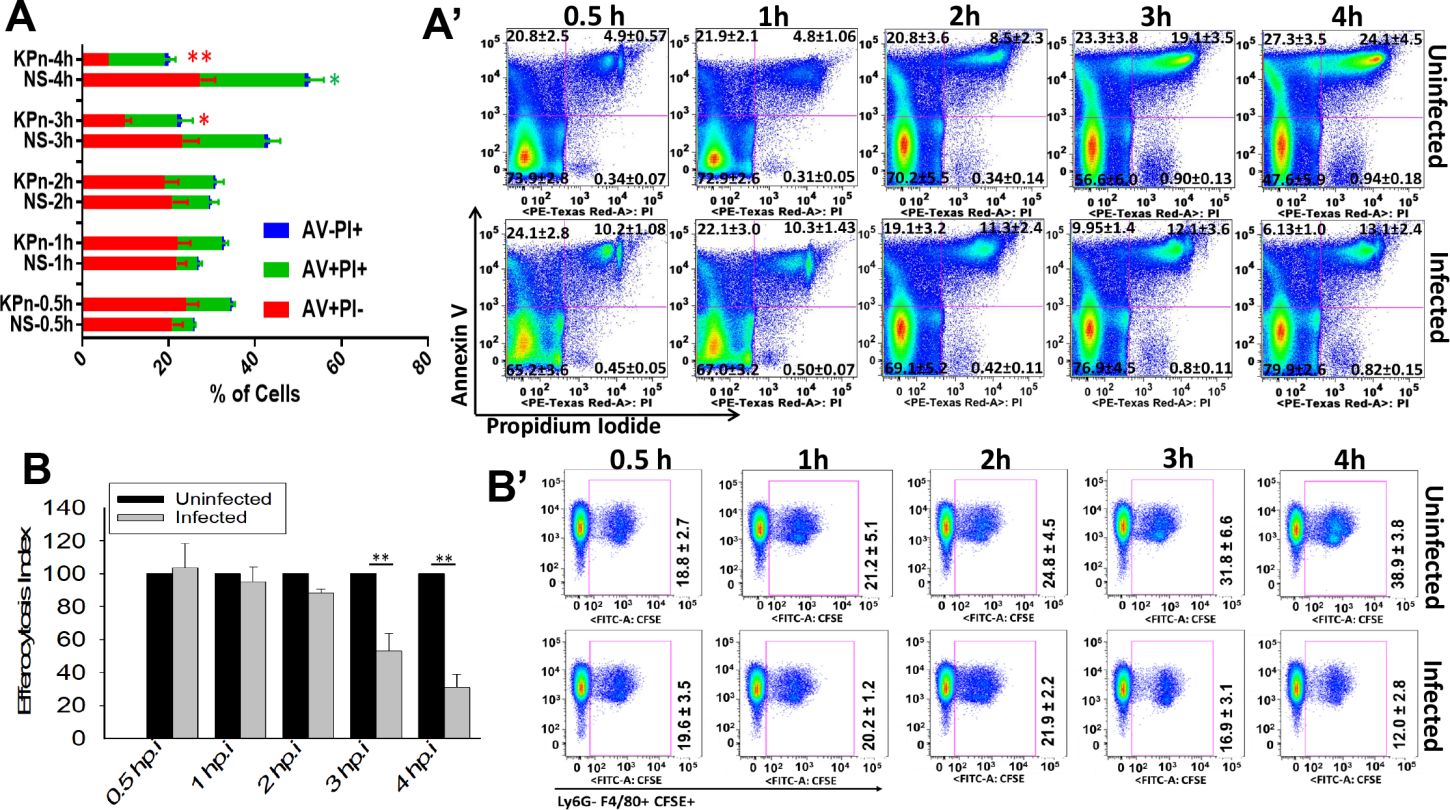
KPn infection decreases the level of surface exposed phosphatidylserine (PS) and reduces efferocytosis of infected neutrophils. **(A)** Neutrophils were left uninfected (NS) or were infected with KPn (MOI 10). The level of PS was monitored by over time post-infection by Annexin V (AV) staining using an Annexin V Apoptosis Detection followed by flow cytometry analysis. Propidium iodide (PI) was used to exclude necrotic cells. Percent of AV+PI- (red bars), AV+PI+ (green bars) and AV-PI+ (blue bars) cells at indicated times is shown (mean ± SEM from 8 independent experiments. Statistical analysis was done by Student’s t test. Red asterisks indicate statistical significance between NS and KPn infected AV+PI- cells and green asterisks indicate statistical significance between NS and KPn infected AV+PI+ cells (*, p<0.05; p**<0.005). **(A’)** shows the representative flow cytometry quadrant plots at each time point. **(B)**. Neutrophils infected for indicated times post-infection were labelled with CFSE and incubated with macrophages for 2hrs followed by flow cytometry to enumerate efferocytic Ly6G-F4/80+CFSE+ macrophages. Efferocytic index was calculated as described above. Data shown are mean ± SEM from 5 independent experiments with 2–3 replicates of each sample per experiments. Student’s t test was used for statistical analysis (p**<0.005). (B’) shows representative dot plots at each time point.

Since PS externalized to the plasma membrane serves as an “eat me” signal for efferocytosis of apoptotic cells by phagocytes [33], we examined if KPn infection modulates PS exposure on infected neutrophils. For this we compared the kinetics of PS externalization in uninfected and KPn infected neutrophils at various times by flow cytometry using Annexin V which specifically binds to PS. Propidium iodide (PI) was used to stain necrotic cells. As shown in Fig 2A and 2A’, approximately 20% of the uninfected neutrophils exhibited surface exposure of PS (Annexin V+PI- cells) after 30 min. of incubation, which increased over a period of 4 hrs, albeit the change over time was not statistically significant, which is typical of spontaneous apoptosis measured during first 4–6 hrs [34]. The KPn infected neutrophils, on the other hand, exhibited similar levels of PS exposure (Annexin V+PI- cells) as their uninfected counterparts up to 2hrs post-infection. However, by 3 hrs post-infection, a drastic reduction in PS exposure was observed in KPn infected neutrophils, as shown by significantly reduced numbers of Annexin V+PI- cells, which remained significantly lower than those in the uninfected cells at 4 hrs (Fig 2A-red bars and 2A’). On the other hand, the numbers of KPn infected late apoptotic (Annexin V+PI+ double positive cells) were significantly reduced at 4hrs, but not 3hrs p.i. (Fig 2A- green bars and 2A’). Together, these data suggested that uninfected neutrophils undergo spontaneous apoptosis indicated by kinetic increase in the numbers of early and late apoptotic cells expressing exofacial PS. KPn infection, on the other hand, arrests this process. Kinetic measurement of efferocytosis of uninfected and infected neutrophils revealed a direct correlation between the reduced PS exposure at 3hrs p.i. with a reduction in their efferocytic uptake, where the uptake of infected neutrophils was reduced by 2–3 fold at 3hrs and 4hrs p.i. compared to the uninfected cells (Fig 2B and 2B’). This suggested that reduced surface exposure of PS on KPn infected neutrophils correlates with a corresponding decrease in their efferocytic uptake by macrophages.

**Fig 3:**
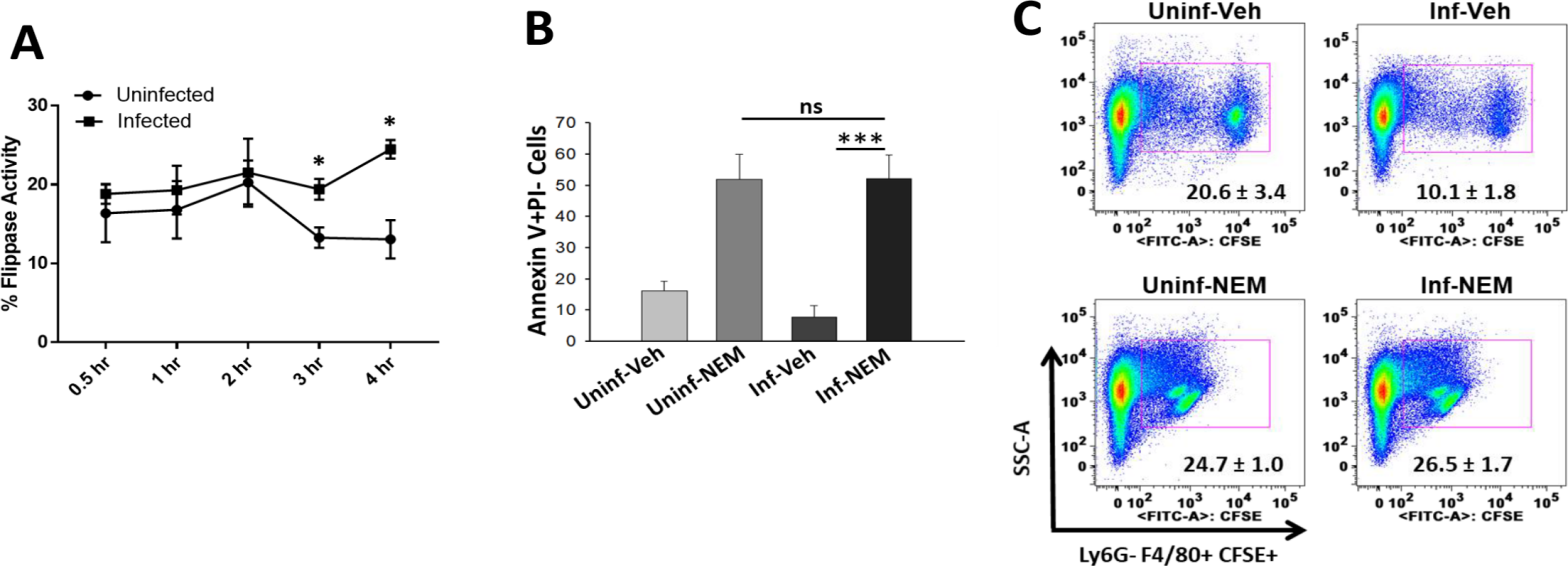
Inhibition of Flippase activity by N-ethylmaleimide (NEM) treatment rescues surface exposed “eat me” signal PS in KPn infected cells and restores efferocytosis. **(A).** KPn infection increases flippase activity in neutrophils. Flippase activity in uninfected and KPn infected neutrophils was calculated at indicated time points using NBD-PS fluorescence before and after exofacial bleaching with sodium dithionite treatment as described in methods. Data shown is mean ± SEM from 3 independent experiments. Student’s t test was used for two group comparisons. (*p<0.05). **(B).** The level of surface exposed PS in uninfected or KPn infected neutrophils with or without NEM treatment (5mM for 30 min) was measured by Annexin V staining followed by flow cytometry. Percent of Annexin V+ PI- cells at 3 hrs post-infection is shown (mean ± SEM from 3 independent experiments. Non-parametric ANOVA with Dunn’s post hoc test was used for statistical analysis (***, p<0.001). **(C)** Uninfected and KPn infected neutrophils were treated with vehicle (ultrapure water) or NEM (5mM for 30 min) as described in Methods, followed by in-vitro efferocytosis. Flow cytometry was performed to enumerate percent of Ly6G- F4/80+ CFSE+ efferocytic cells. Representative dot plots from one experiment out of 3 independent experiments is shown. The numbers on dot plots are mean ± SEM from 3 independent experiments with 3–4 replicates per experiment for each sample. No statistically significant difference was found between efferocytosis of uninfected and KPn infected neutrophils upon NEM treatment. Uninf; Uninfected, Inf; KPn infected, Veh; Vehicle, NEM; N-ethylmaleimide.

Flippases translocate PS from outer to inner plasma membrane leaflet in live cells and apoptotic PS exposure is accompanied by loss of flippase activity [35]. In order to understand the mechanism underlying the reduced exofacial PS exposure in KPn infected neutrophils, we examined flippase activity in these cells by measuring the internalization 1-oleoyl-2-{6-[(7-nitro-2-1,3-benzoxadiazol-4-yl)amino]hexanoyl}-sn-glycero-3-PS (NBD-PS). As shown in Fig 3A, the phospholipid flippase activity was significantly higher in KPn infected neutrophils compared to their uninfected counterparts at 3hrs and 4hrs p.i. This coincided with reduced PS exposure observed at these time points shown in Fig 2A. To confirm that increased flippase activity and concomitant reduced PS exposure is responsible for retarded efferocytosis of KPn infected neutrophils, we treated the cells with flippase inhibitor N-ethylmaleimide (NEM), which triggers flipping of PS to outer plasma membrane of cells [36–38]. Indeed, treatment with NEM dramatically increased the PS exposure on these KPn infected neutrophils to similar levels as observed in uninfected neutrophils (Fig 3B). As compared to 9.9% of the untreated KPn infected neutrophils, 52.1% ± 7.7% of KPn infected cells bound Annexin V after NEM treatment (Fig 3B). Having established that NEM treatment increased the surface exposed PS in KPn infected neutrophils, we next tested if efferocytic uptake of KPn infected neutrophils was improved following NEM treatment. As shown in Fig 3C, reversal of PS externalization upon NEM treatment restored the efferocytic uptake of KPn infected neutrophils (26.5 ± 1.7%) to the levels observed with uninfected neutrophils (24.7 ± 1.0%). Together these data showed that KPn infection likely causes increased flipping of PS resulting in its diminished externalization and thus an impairment of PS-dependent recognition and efferocytic uptake of infected neutrophils by macrophages.

**Fig 4:**
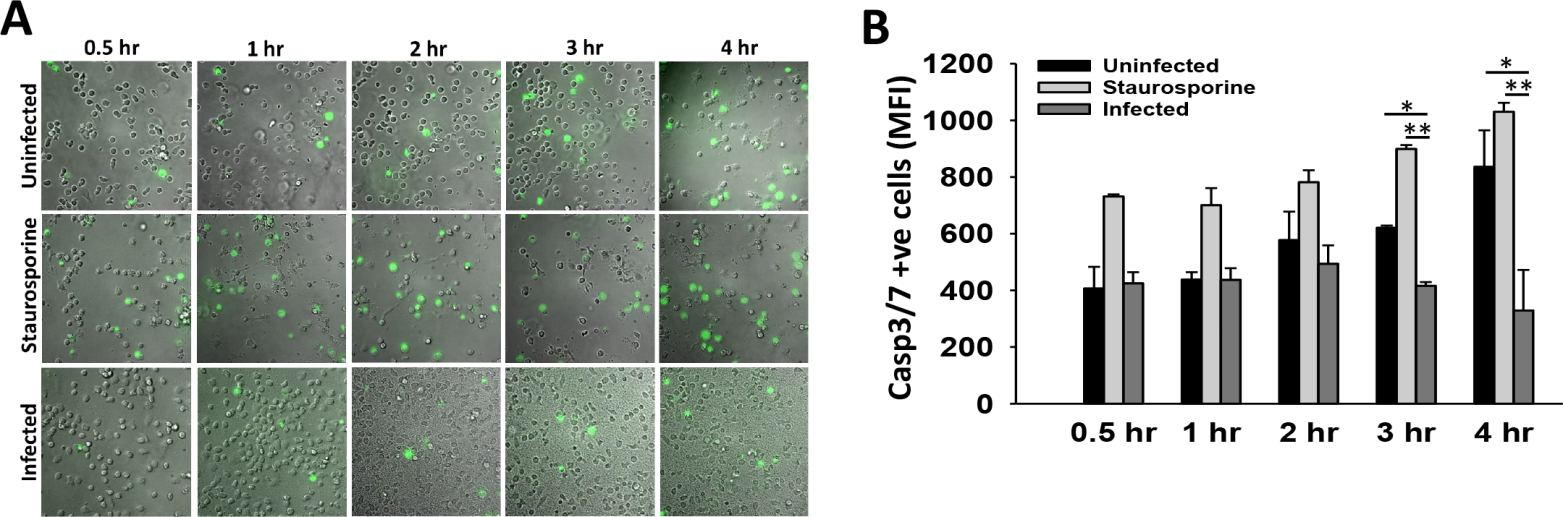
Reduced Caspase3/7 activation in KPn infected neutrophils. **(A).** Representative images of uninfected, KPn infected or Staurosporine treated cells showing Caspse3/7 activation (green) by using CellEvent Caspase-3/7 Green Detection Reagent as described in methods. **(B).** Mean Fluorescence intensity (MFI) of cells with activated Caspase3/7 over time as measured by flow cytometry using Caspase3/7 activation kit. Data shown are mean ± SEM from 3 independent experiments with 2–3 replicates per group. Statistical analysis was done by ANOVA with Dunn’s post hoc analysis (*p<0.05, p**<0.005).

Exofacial PS is a characteristic feature of apoptosis [35]. Our observation of reduced PS exposure and increased flippase activity in KPn infected neutrophils prompted us to examine apoptosis activation in these cells. Since executioner caspases -3 and -7 are universally activated during apoptosis and have also been shown to inactivate flippases to facilitate PS externalization [39, 40], we performed live cell imaging to compare caspase3/7 activation in uninfected and KPn infected neutrophils at various times post-infection. Neutrophils treated with apoptosis inducer staurosporine served as experimental positive control which showed increased activation of Caspase-3 and -7 over time as determined by fluorogenic cleavage of CellEvent Caspase-3/7 Green Detection Reagent (DEVD peptide) by activated caspases (Fig 4A, middle panel). Uninfected neutrophils also exhibited an increase in caspase activation albeit to a lesser extent than the staurosporine treatment, indicating spontaneous apoptosis, characteristic of primary neutrophils (Fig 4A upper panel). In contrast, significantly fewer neutrophils exhibited Caspase 3/7 activation upon KPn infection, as compared to the uninfected neutrophils or the positive control (Fig 4A, lower panel). Quantitation of the mean fluorescence intensity by flow cytometry analysis confirmed these observations and showed a significantly lower fluorescence detection at 3hrs and 4hrs post-infection in KPn infected neutrophils, compared to the uninfected or staurosporine treated neutrophils (Fig 4B). Taken together, these data suggested that KPn infection reduces activation of Caspases 3/7 which likely spares flippase activity to allow increased retention of PS to the inner leaflet of plasma membrane eventuating in impaired recognition and efferocytosis of these cells by macrophages.

### KPn infection activates necroptosis machinery in neutrophils

**Fig 5:**
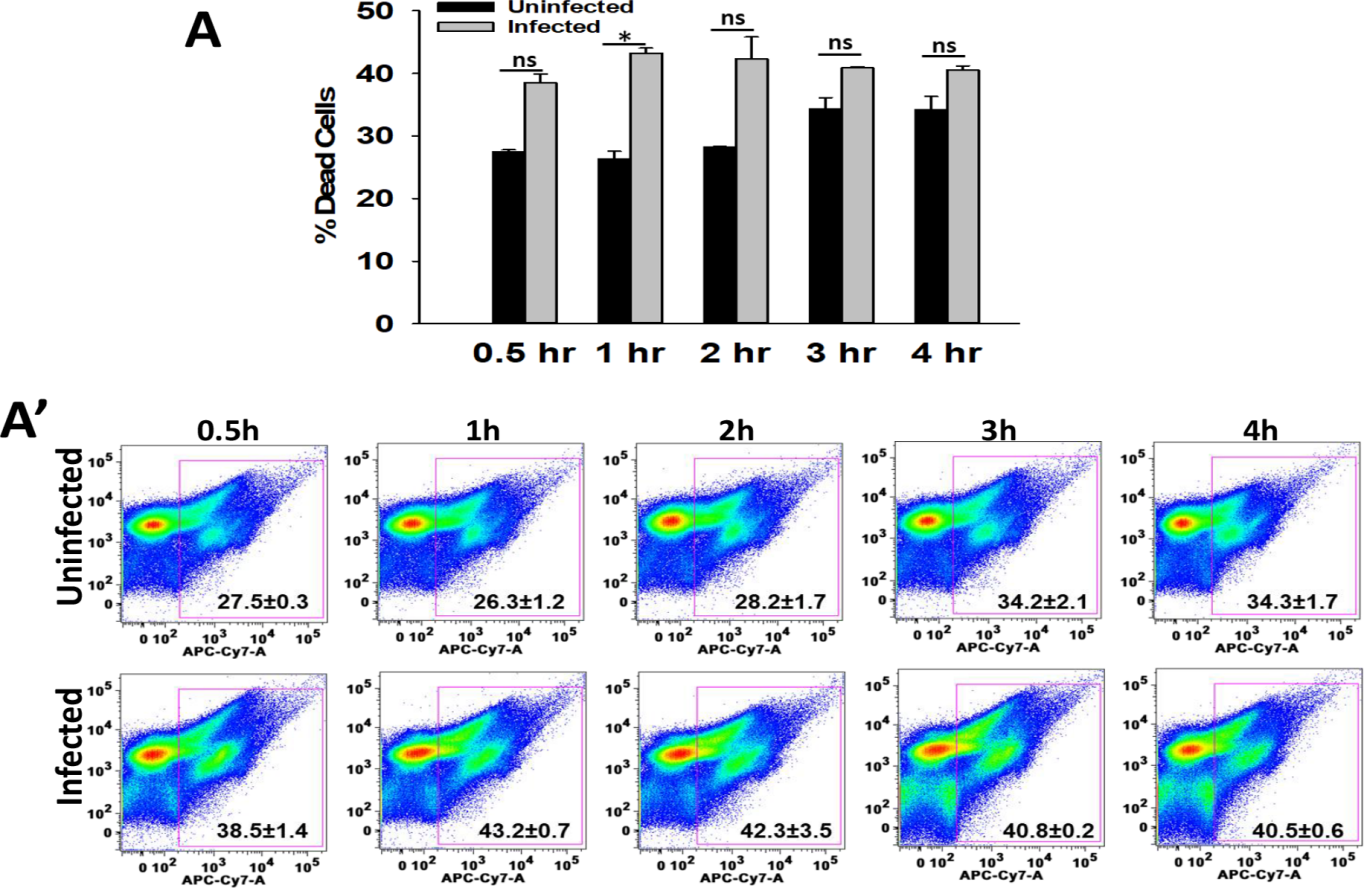
KPn infection causes cell death. Peritoneal neutrophils uninfected or infected with KPn (MOI 10). At indicated times, dead cells were enumerated by staining with LIVE/DEADFixable Near-IR Dead Cell Stain Kit (Invitrogen) per manufacturer’s instructions. Cells were then washed and analyzed by flow cytometry. Data in (A) is mean ± SEM from 2 independent experiments with 3–5 replicates each time. Representative dot plots are shown in (A’). Student’s t test was used for statistical analysis (p*<0.05). ns, not significant.

**Fig 6:**
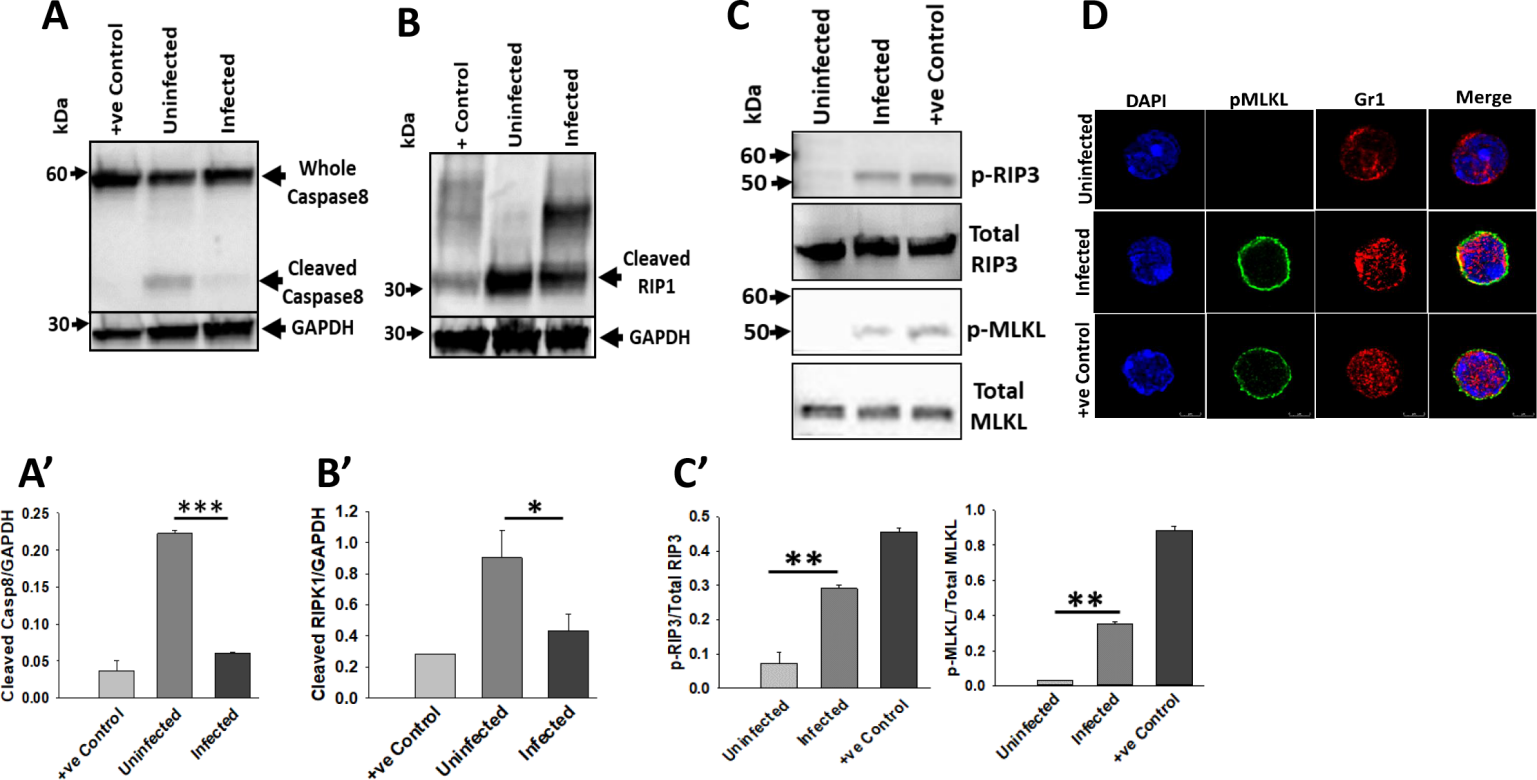
KPn infection activates necroptosis in neutrophils. Representative immunoblots for cleaved caspase-8 **(A)** and RIPK1 **(B)**, as well as phosphorylated RIPK3 and MLKL **(C)** in whole cell lysates from uninfected and KPn infected neutrophils (3hp.i.) are shown. Lysate from primary neutrophils treated with TNF-α with SMAC mimetic and pan-caspase inhibitor Q‐VD-OPh were used as positive control for necroptosis activation. Densitometry analysis of the blots was preformed using Image J software and band intensities were represented as **(A’ and B’)** ratio of the protein of interest and the internal control levels; and **(C’)** ratio of phosphorylated protein and the total protein. Data shown are mean ± SEM from 3–4 independent experiments. Statistical analysis was done by ANOVA with Dunn’s post hoc analysis (*, *p*<0.05; p**<0.005***, *p*<0.001). **(D).** Representative confocal microscopy images of uninfected, KPn infected (MOI 10 for 3 hrs) and neutrophils treated with TNF-α with SMAC mimetic and pan-caspase inhibitor Q‐VD-OPh (necroptosis activation). The cells were imaged for translocation of phosphorylated MLKL (green) to neutrophil membrane. Gr1 (red) was used as neutrophil membrane marker. Nuclei were stained with DAPI. Scale bar 5μM. The experiment was repeated twice with same results.

Despite the impediment of apoptosis as evident by an impaired caspase 3/7 activation and reduced PS exposure, we found a higher number of KPn infected neutrophils stained positive with an amine-reactive dye, at 3hrs and 4hrs p.i. as compared to Annexin V+PI- cells with PS exposure at that time (Fig 5). This staining method captures global cell death without discriminating between various cell death types. This data suggested that instead of inhibiting cell death, KPn infection likely reprograms the cell death modality. To test this hypothesis, we analyzed necroptosis activation in uninfected and KPn infected neutrophils. For this, we first examined caspase 8 which is centrally positioned to control the extrinsic pathway of apoptosis by activating caspase 3 and 7 and by degradation of RIPK1 [41, 42]. Western blot analysis of Caspase 8 in uninfected and KPn infected neutrophils showed a significantly reduced processing of procaspase 8 into active Caspase 8 in the infected neutrophils (Fig 6A and 6A’). This coincided with the reduced degradation of RIPK1 in KPn infected neutrophils as compared to their uninfected counterparts, indicating inhibition of apoptosis and activation of necroptosis (Fig 6B and 6B’). Indeed, phosphorylation of RIPK3 as well as MLKL (Fig 6C and 6C’) was significantly higher in KPn infected neutrophils as compared to the uninfected cells strongly suggesting activation of necroptosis in these cells upon infection. As a positive control, neutrophils treated with TNF-alpha in combination with SMAC mimetic and caspase inhibitor Q-VD-OPh were analyzed for necroptosis activation in parallel with all samples (Fig 6 +ve control). A hallmark of necroptosis activation is the translocation of MLKL oligomers to the plasma membrane, following RIPK3-mediated phosphorylation of MLKL [43]. Confocal imaging of uninfected and KPn infected neutrophils at 3hrs post-infection showed a strong phospho-MLKL signal on the plasma membrane of infected neutrophils which was similar to that observed with the positive control cells (Fig 6D). Together with the western blot analysis, these data strongly suggested that KPn infection skews the cell death in neutrophils toward necroptosis and away from apoptosis, a cell death mode favorable for efferocytic clearance.

### Inhibition of necroptosis rescues the efferocytic uptake of KPn infected neutrophils *in-vitro*

**Fig 7:**
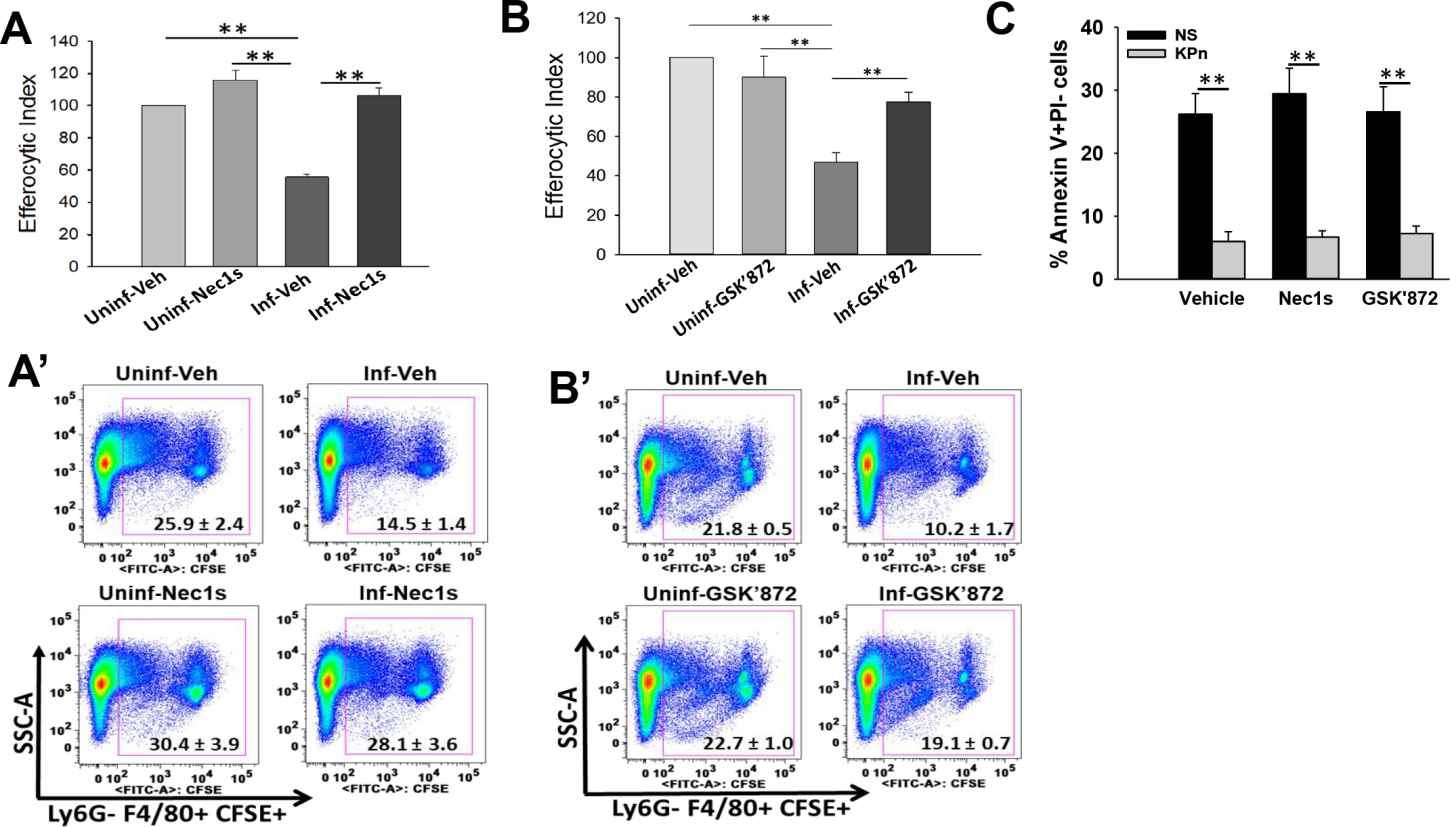
Necroptosis blockage restores the efferocytosis of KPn infected neutrophils independent of PS exposure. **(A).** Efferocytosis was performed with CFSE-labelled uninfected or KPn infected neutrophils treated with RIPK-1 inhibitor Necrostatin-1s or vehicle alone as described in methods. Efferocytic index was calculated as percent efferocytotic cells (Ly6G- F4/80+ CFSE+ macrophages) with infected neutrophils normalized to those with uninfected cells taken as 100%. **(A’)** shows representative dot plots from one of these experiments. The numbers shown are average percentage ± SEM from 3–5 independent experiments with 3–4 replicates. (**B & B’)** show similar experiment with RIPK3 inhibitor GSK’872 treatment. **(C).** Uninfected or KPn infected neutrophils were treated with vehicle alone (DMSO) or Nec1s or GSK’872 as described in methods followed by flow cytometry analysis of PS levels (Annexin V+PI- cells) at 3hp.i. using an Annexin V Apoptosis Detection kit. Statistical analysis was done by ANOVA with Dunn’s post hoc analysis (**, p<0.005).

To establish the physiological relevance of modulation of cell death to necroptosis by KPn, we examined the effect pharmacological inhibition of necroptosis on efferocytosis of neutrophils. For this we treated uninfected and KPn infected neutrophils with Necrostatin 1s (Nec1s), a specific RIPK1 inhibitor shown to suppress necroptosis [30]. As shown in Fig 7, a significantly lower percentage of KPn infected neutrophils were efferocytosed as compared to the uninfected cells. Pretreatment with Nec1s reversed this effect of KPn infection and restored the efferocytic uptake of infected neutrophils to the levels similar to uninfected cells (Fig 7A and 7A’). Of note, Nec1s treatment did not significantly alter the efferocytosis of uninfected neutrophils undergoing spontaneous apoptosis. To further confirm the role of necroptosis in KPn infection-driven efferocytosis inhibition, we also tested the effect of RIPK3 inhibitor GSK’872. Indeed, GSK’872 treatment of KPn infected neutrophils significantly increased their efferocytic uptake in comparison with the cells treated with the vehicle alone (Fig 7B and 7B’). The treatment of infected neutrophils with Nec1s or GSK’872 did not increase PS exposure in these cells (Fig 7C). This indicated that activation of necroptosis and modulation of PS exposure are likely two independent mechanisms underlying the impaired effrocytosis of KPn infected neutrophils.

### Inhibition of necroptosis improves disease outcome in KPn pneumonia

**Fig 8:**
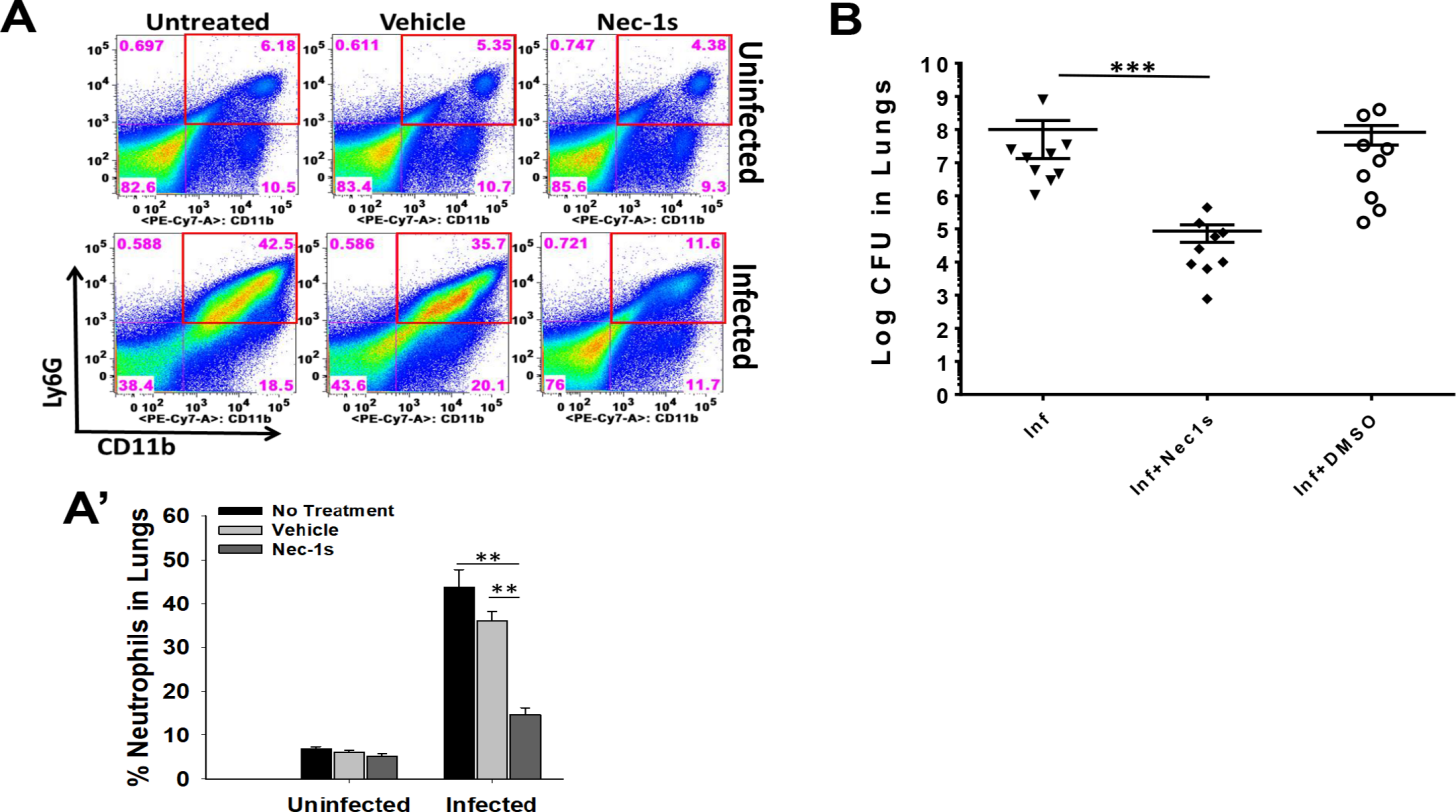
Necroptosis blockage improves the disease outcome in KPn pneumonia. **(A).** C57BL/6 mice infected intranasally with 3.0x10^4^ CFUs of KPn received intraperitoneally 100μl of 100μM necrostatin-1s or vehicle (DMSO) 2hrs prior to infection and then every 4hrs for 12hrs post-infection. Mice were sacrificed at 3 days post infection, lungs were isolated and were processed for flow cytometry analysis of neutrophils by staining with anti-Ly6G-APC and anti-CD11b-Pacific Blue antibodies. Representative dot plots from one out of 3 independent experiments is shown. **(A’)** shows percent neutrophils (Ly6G+CD11b+) as mean ± SEM from three independent experiments with 3–5 mice per group in each experiment. Statistical analysis was done by ANOVA with Dunn’s post hoc analysis (**, p<0.005). **(B).** In separate set of experiments performed similarly as in (A), lungs were homogenized aseptically and plated to enumerate bacterial burden. Each dot represents one mouse. *n* = 9 in each group in 3 independent experiments. (***, p<0.001).

We found that efferocytosis of KPn infected neutrophils is impaired in the lungs of mice (Fig 1D). Having established necroptosis as one of the underlying mechanisms, we sought to examine the effect of necroptosis inhibition by Nec1s treatment on the disease outcome in mice undergoing KPn pneumonia. For this, we treated mice 2hrs before infection and every 4hrs up to 12hrs post-infection with Nec1s as described [44]. Flow cytometry analysis of lungs at 3dp.i., the septic phase of infection exhibiting peak neutrophil accumulation [11, 14, 45], showed that the number of CD11b+Ly6G+ neutrophils was significantly reduced in KPn infected mice upon Nec1s treatment as compared to untreated or vehicle treated KPn infected mice (Fig 8A and 8A’). This indicated that neutrophilia was attenuated by necroptosis inhibition. The bacterial titers in the lungs of Nec1s treated mice was also significantly reduced as compared to the untreated or vehicle treated animals (Fig 8B), further supporting the protective effect of necroptosis inhibition. Overall, these data showed that necroptosis has pathological consequences in promoting pneumonia during KPn infection and inhibition of this pathway improves the disease pathology.

## Discussion

During an acute injury, neutrophils are promptly recruited to the site which, upon the resolution of insult, undergo apoptosis and are cleared by efferocytosis without inducing overt inflammation [9]. Apoptotic cells are swiftly removed by efferocytic cells by recognition of surface “eat me” signal phosphatidylserine (PS), exposed as a result of caspase-mediated inhibition of flippase activity on apoptotic cell membrane [46]. Here we report that *Klebsiella pneumoniae*, an opportunistic pathogen, prevents efferocytic clearance of neutrophils by reducing surface exposure of PS via modulation of flippase activity. We also show that KPn infection skews the programmed cells death away from “efferocytosis favorable” apoptosis toward necroptosis, which is likely the associated with increased neutrophilia and poor disease outcome. Concomitantly, reversal of PS exposure and blockage of necroptosis improves efferocytic clearance of neutrophils as well as disease outcome in murine inhalation model of KPn pneumonia. Our study provides important insights into pathogenic mechanisms that can be targeted for future antimicrobial therapies for this infection.

Neutrophils are short-lived cells that undergo constitutive or spontaneous apoptosis following which these cells are cleared by phagocytes via efferocytosis, to prevent secondary necrosis and release of their noxious cargo that may cause bystander tissue damage. Induction of apoptosis, typically characterized by activation of executioner caspases and externalization of phosphatidylserine (PS), is thus considered the first step of initiation of efferocytosis process. Efferocytic phagocytes recognize specific “eat-me” signals on apoptotic cells in a receptor dependent manner [33]. Exofacial PS is considered the most well-characterized “eat-me” signal recognized by the phagocytes for removal of apoptotic cells [47]. Owing to an efficient and immunologically silent nature of this event, many viruses and parasites have been reported to use PS mimicry by concentrating PS on their surface and hijack efferocytic machinery of host cells to promote their internalization and cell-to-cell spread [48–50]. In contrast to this, pathogen -mediated skewing of efferocytic clearance of immune cells via PS recognition is much less studied. *Staphylococcus aureus* is shown to inhibit efferocytic clearance of neutrophils via upregulation of CD47, a “don’t eat me” signal and an alternative cell death pathway of necroptosis [51]. In our study we instead found that KPn infection results in a drastic down modulation of exofacial PS, reduced apoptotic caspases and increased activation of necroptosis machinery. The relevance of these events was confirmed by reversal of PS exposure and inhibition of necroptosis on KPn infected neutrophils, which rescued their efferocytic clearance *in-vitro* and reduced the bacterial burden as well as neutrophilia *in-vivo* in murine pneumoseptic KPn infection. Although delayed apoptosis in KPn infected neutrophils [52, 53]; and indirect evidence of necroptosis activation in KPn infected macrophages [32] was recently reported, we present the first evidence of the mechanism underlying reduced PS exposure; and physiological relevance of KPn mediated skewing of cell death modality in neutrophils to avoid their efferocytic clearance. PS-dependent clearance of immune cells results in the release of anti-inflammatory and pro-resolving factors which downregulate the inflammation [54]. In addition, efferocytic uptake of apoptotic neutrophils regulates granulopoiesis and peripheral neutrophilia via an IL-23/IL-17/G-CSF axis [55]. On the other hand, neutrophils undergoing necroptosis are less amenable to efferocytic clearance and amplify inflammation [56, 57]. It is tempting to speculate that reduced activation of apoptosis and PS exposure in KPn infected neutrophils, in combination with activation of necroptosis machinery, likely result in the loss of the negative regulatory axis thus contributing to neutrophilia and inflammation characteristic of this infection. Studies to examine this line of inquiry are currently underway in our laboratory.

PS distribution on the lipid bilayer in biological membranes is controlled by the activity of ATP-dependent flippases and scramblases [58]. Flippases are responsible for removing PS from the external leaflet by ATP-dependent active transport; the relevant proteins are members of the type IV subfamily of P-type ATPases. Constant surveillance of the external leaflet by these enzymes establishes a normal plasma membrane distribution in which virtually all of the PS is in the inner leaflet. The second enzymatic activity termed scramblases, regulating the distribution of PS between leaflets catalyzes rapid and nonspecific exchange of phospholipids between the two sides of the bilayer. In the apoptotic caspase cascade, caspases 3 and 7 cleave the flippases to inactivate them leading to irreversible PS exposure [35]. We found a significantly reduced caspase 3 and 7 activation in neutrophils upon KPn infection, suggesting that KPn mediated impairment of PS exposure involves modulation of flippase activity. Indeed, KPn infected neutrophils exhibited enhanced flippase activity, which likely contributes to restriction of PS to inner leaflet resulting in its reduced exofacial exposure. While the involvement of scramblases and the identity of specific flippase is an ongoing area of investigation in our laboratory, our results show, for the first time, that KPn downregulates PS exposure on neutrophils by modulating phospholipid translocase activity.

Necroptosis is defined as a caspase-independent non-apoptotic form of regulated cell death which requires RIPK1 and RIPK3-mediated activation of pseudokinase, mixed lineage kinase-like protein (MLKL) [59]. Extrinsic pathway of apoptosis activated by death ligands such as FasL or TLR ligands promotes processing of caspase-8 which degrades RIPK1 and drives apoptotic cell death [60]. Conversely, inhibition of caspase 8 spares RIPK1 to promote RIPK3 autophosphorylation and recruitment of MLKL to form a necroptosome complex [61]. Phosphorylation of the C-terminal pseudokinase domain of MLKL by RIPK3 promotes MLKL translocation to plasma membrane, which eventually disrupts the membrane integrity [62]. Our results showed reduced processing of caspase 8 in KPn infected neutrophils indicating the apoptosis arrest in these cells. This coincided with increased RIPK1 levels, RIPK3 phosphorylation and MLKL phosphorylation as well as translocation of phospho-MLKL to plasma membrane, strongly suggesting the activation of necroptosis pathway in KPN infected neutrophils. This supports the notion that KPn drives cell death away from immunologically silent apoptosis which favors efferocytosis, toward inflammatory necroptosis [57]. We show the physiological consequence of this phenomenon by increased efferocytic uptake of infected neutrophils upon treatment with NEM to increase PS exposure as well as with inhibitors of necroptosis. The observation of increased global cell death at 3hrs in KPn infected neutrophils (Fig 5) as compared to much lower early and late apoptotic/necrotic cells at that time point (Fig 2) is intriguing. This may be reflective of higher sensitivity of amine-binding dyes compared to propidium iodide, or that there may be a certain interval/time lag between the MLKL localization and lysis of cells. As such, the precise events following integration of MLKL into plasma membrane leading up to plasma membrane rupture are still poorly understood. Whether the increased RIPK1, RIPK3 and MLKL activation marks a typical necroptotic, lytic event during infection or if KPn utilizes the necroptosis machinery to modulate cellular functions in its favor, or a combination of both, requires further investigation. Nevertheless, our report presents convincing evidence that necroptosis machinery is activated upon KPn infection which, along with reduced PS exposure via increased flippase activity, contributes to reduced efferocytic clearance of neutrophils.

Necroptotic fibroblasts and monocytic cell lines were recently reported to expose PS on their surface in pMLKL-dependent fashion [63, 64]. This elegant study implicated plasma-membrane associated active MLKL in rapid PS exposure that occurs within 5 minutes of RIPK3 and MLKL activation. In our study we found a reduced PS exposure, but increased pMLKL in primary neutrophils after 3 hrs of infection with bacterial pathogen KPn. PS exposure was increased in KPn infected neutrophils early during infection (30 min). However, we did not find activated RIPK3 or p-MLKL at that time in uninfected or infected neutrophils. This suggests that different pathways of PS exposure and necroptosis are activated in primary neutrophils in response to bacterial infection versus the fibroblasts and monocytic cell lines activated by different stimuli used in the reported study. This is further supported by our observation that inhibition of necroptosis does not restore PS exposure in infected neutrophils. In light of our results showing the reversal of efferocytosis inhibition upon increased PS exposure as well as upon inhibition of necroptosis, it is highly likely that KPn utilizes two independent strategies to inhibit the clearance of neutrophils and to drive the neutrophilia and inflammation characteristic of pneumonic infection. Improved disease outcome in terms of reduced bacterial burden and decreased neutrophil accumulation in lungs of pneumonic mice, upon blockage of necroptosis supports a detrimental effect of cell death modulation by this pathogen. Our results are in line with elegant studies showing the pathological effect of necroptosis in development of bacterial pneumonia and possibility of using necroptosis inhibitors as an adjunct therapy for bacterial infections [30, 32, 44, 65].

KPn has recently gained attention as a “successful” pathogen owing to an emergence of hypervirulent strains as well as antibiotic resistance [66]. The wide range of infections caused by this pathogen in immunocompromised and immune-competent individuals have become increasingly difficult to treat owing partly to the arsenal of virulence factors exhibited by this pathogen that it utilizes to protect itself from host immune response [67]. Based on these virulence factors KPn has been categorized as an “evader” rather than an “offender” pathogen. Our results presented here provide new insights into the strategies employed by this pathogen to actively suppress an important host defense i.e. efferocytic clearance of neutrophils by modulation of cell death pathway and PS externalization. Although the identity of putative virulence factor/s involved in the process are currently being worked out in our lab, our data showing downregulation of PS exposure by increased flippase activity, reduced activation of apoptosis executioner caspases and reprogramming of cell death toward inflammatory necroptosis pathway in neutrophils infected with KPn, highlight virulence strategies employed by this pathogen to impair efferocytic clearance of these cells by macrophages. Given its host protective consequences, subversion of efferocytosis may be advantageous for this pathogen to establish infection. Also of importance in this regard, a recent report showed neutrophils as vehicles for KPn in its dissemination to establish liver abscess, a severe clinical complication of KPn infection [16]. Efferocytic clearance of KPn-infected neutrophils thus may aid in circumventing these distant metastatic complications, as has been shown in case of mycobacterial infection [22]. Notwithstanding the identity of virulence factors involved, our findings open new avenues to treat and prevent the systemic spread of this infection. As antibiotic resistance is a serious problem associated with *Klebsiella* infections, elucidation of mechanism by which this pathogen manipulates efferocytosis, as reported here, might provide novel therapeutic targets.

## Materials and methods

### Bacterial strains and mice

The KPn (ATCC strain 43816) were grown to log phase in LB medium at 37°C. For isolation of cells and *in-vivo* experiments 6–8 weeks old wild-type C57BL/6 bred in the animal facility of the University of North Dakota were used. The animals were handled according to the institutional and federal guidelines.

### Ex-vivo efferocytosis

Peritoneal neutrophils and macrophages were isolated 12-16hrs and 5 days respectively after intraperitoneal injection of sterile 4% thioglycollate (BD Biosciences, San Jose, CA) (10, 12). Purity of the cells was ascertained by flow cytometry analysis (Ly6G+ neutrophils 80–85%; F4/80+ macrophages 85–90%). Isolated neutrophils were left uninfected or infected with 10 MOI of KPn for 3 hrs followed by labelling with Carboxyfluorescein succinimidyl ester (CFSE; CellTrace CFSE Cell Proliferation Kit from Invitrogen). Macrophages seeded on 6 well plates (0.5x10^6^ cell/ml) were incubated with CFSE-labelled uninfected or KPn infected neutrophils at a ratio of 5:1. After 2hrs of efferocytosis, non-internalized neutrophils were removed by washing thoroughly. Macrophages were scraped and stained with F4/80 and Ly6G antibodies for flow cytometry. Gating scheme to quantitate Ly6G-F4/80+CFSE+ efferocytic macrophages that had internalized labelled neutrophils is shown in Fig 1A. For some experiments, uninfected and infected neutrophils were pretreated with 5 mM of N-ethylmaleimide (NEM) or with necroptosis inhibitors necrostatin-1s (100 μM) (BioVision, California) or GSK’872 (3 μM) (Millipore Sigma, St. Louis, MO) for 30 min before infection. For calculating efferocytic index, the percentage of Ly6G-F4/80+CFSE+ macrophages engulfing KPn infected neutrophils was normalized to those that engulfed uninfected neutrophils (taken as 100%) [68, 69].

### In-vivo efferocytosis

Uninfected or KPn infected (MOI 10) peritoneal neutrophils labelled with CFSE (repeated) were administered intranasally (35μL/3.5x10^6^ cells) into mice anaesthetized using a mixture of 30mg/ml ketamine and 4 mg/ml xylazine in PBS. Lungs were lavaged 2 hrs after the instillation as described by us [11, 13]. Efferocytic uptake by macrophages was quantitated by flow cytometry using PE-Cy7 conjugated anti-F4/80 and APC conjugated anti-Ly6G antibodies (BioLegend, San Diego, CA) to enumerate Ly6G- F4/80+ CFSE+ alveolar macrophages. Efferocytic index was calculated as described above.

### Flow cytometry analysis of PS exposure and cell death

Peritoneal neutrophils were left uninfected or infected with 10 MOI of KPn for 3 hrs. A FITC Annexin V Apoptosis Detection Kit (BD Biosciences, San Jose, CA) was used according to manufacturer’s instructions followed by flow cytometry analysis to enumerate percentage of Ly6G+ PI- Annexin V+ cells with surface exposed PS using BD LSR II flow cytometer (Becton Dickinson, San Jose, CA). To enumerate global cell death, neutrophils uninfected or infected with *K*. *pneumoniae* for various times as described above were stained using LIVE/DEAD Fixable Near-IR Dead Cell Stain Kit (Invitrogen) in serum free PBS for 30 minutes on ice per manufacturer’s instructions. Cells were then washed with PBS containing 1% FBS and analyzed by flow cytometry. For caspase 3/7 activation staining, CellEvent Caspase-3/7 Green Detection Reagent (Invitrogen) kinetic assay was used according to the manufacturer’s instructions. Briefly, peritoneal neutrophils were loaded with optimized concentration of the detection reagent for 30 minutes at 37°C. Cells were then either left uninfected or infected with *K*. *pneumoniae* (at 10 MOI) in complete RPMI medium. At indicated times, uninfected and infected cells were analyzed for Caspase3/7 activation by flow cytometry to quantify Mean Fluorescence intensity (MFI) or were imaged using Olympus upright phase-contrast fluorescent microscope. Staurosporine (5 μM, Sigma-Aldrich), an apoptosis inducer was used a positive control for each time point.

### Measurement of flippase activity

Flippase activity in uninfected and KPn infected neutrophils was determined as described previously [70, 71]. Briefly, peritoneal neutrophils were incubated with 1 μl of 1 mg/ml of 1,2-dioleoyl-sn-glycero-3-phospho-L-serine-N-(7-nitro-2-1,3-benzoxadiazol-4-yl) (NBD-PS) in 1 ml HBSS (Hank’s balanced Salt Solution) containing 1 g/liter glucose for 20 min at 37°C. Cells were then washed, pelleted and suspended in the RPMI-1640 without or with *K*. *pneumoniae* (10 MOI) for various time points. After each incubation period, neutrophils were washed with 1 ml PBS containing 5% fatty acid free BSA. To measure the NBD-PS in the inner leaflet of the membrane, 40 μl of freshly prepared 1 M sodium dithionite prepared in 0.5 M Tris was added to sample, in order to bleach the NBD-PS on the outer leaflet. Mean fluorescence intensity (MFI) of KPn infected and uninfected neutrophils was then measured by flow cytometry. Flippase activity of each sample was quantified by comparing NBD-PS fluorescence before F(o) and after F(x) bleaching according to the following Equation.

$$Fflip=\frac{F(0)}{F(x)}$$

The percent Flippase activity was calculated using the following equation where F_flip (100)_ is the NBD-PS stained cells.

Flip[%]=(Fflip÷Fflip(100))∙100

### Analysis for necroptosis activation

For detection of necroptosis markers, peritoneal neutrophils uninfected or infected with 10 MOI KPn for 3 hours were lysed with RIPA buffer containing protease and phosphatase inhibitors (Sigma) as described previously by us and others [44, 72, 73] and probed with anti-mouse Caspase 8, RIPK1 antibodies (Cell Signaling Technology, Danvers, MA) and phosphor-RIP3 S232 & T231, phosphor-MLKL S345 antibodies (Abcam, Cambridge, MA) by immunoblot analysis. Glyceraldehyde 3-phosphate dehydrogenase (GAPDH) was used as loading control. Peritoneal neutrophils treated with 1 μM SMAC mimetic AT‐406 and 20 μM Q‐VD-OPh for 30 min prior to stimulating with 100 ng/mL mouse TNF-α were used as positive control for necroptosis induction [74]. Densitometry analysis on blots was performed using Image J software and band intensities were represented as the ratio of the protein of interest and the internal control levels. For phosphorylated proteins, the band intensities were represented as the ratio of phosphorylated and total protein.

For plasma membrane localization of phospho-MLKL, uninfected, infected or positive control neutrophils were washed with PBS and were fixed on Poly-L-lysine coated glass slides with 4% paraformaldehyde. Cells were then processed for immunostaining as described by us [11, 14, 15, 75] using mouse monoclonal anti-phospho-MLKL (0.1μg/ml) (Millipore Sigma) conjugated to Alexa Fluor 488 (Molecular Probes, Eugene, OR) and rat monoclonal anti-Gr1 (eBioscience) followed by goat anti-rabbit Alexa Fluor 546 secondary antibody. Plasma membrane localization of proteins was analyzed using Zeiss LSM 510 meta confocal microscope and images were processed using ImageJ software.

### Infection of mice

Mice were anaesthetized with a mixture of 30mg/ml ketamine and 4 mg/ml xylazine in PBS and were infected intranasally with 3.0 x 10^4^ CFUs in 20μl of saline, of KPn or with 20 μl of saline alone. In some instances, mice received intraperitoneal injections (100μl) of necrostatin-1s (100μM) or vehicle alone (DMSO) 2hrs before infection and every 4hrs up to 12hrs post-infection as described [44]. No toxicity or overt pathology was observed at this dose. The mice were euthanized at 3dp.i. and lungs were aseptically homogenized in cold PBS with Complete protease inhibitor cocktail (Roche Diagnostics, Germany). For the bacterial burden analyses, serially diluted homogenates were plated on LB agar and incubated at 37°C overnight.

For enumeration of neutrophils in mouse lungs, lungs cells were harvested from mice at 3 days p.i. and processed as previously described by us [11, 14, 45]. Quantitation of neutrophils by flow cytometry (using a BD LSR II, Becton Dickinson, San Jose, CA) was done by quantitating Ly6G+CD11b+ cells stained with Pacific Blue anti-mouse CD11b (Clone M1/70) and APC anti-mouse Ly6G (Clone 1A8) antibodies (Biolegend, San Diego, CA). FlowJo (Tree Star) software was used to analyze the data.

### Statistical analysis

Statistical analyses were performed using the Student t test (SIGMA PLOT 8.0, Systat Software, San Jose, CA) for two-group comparisons. A *p* value of ≤ 0.05 was considered statistically significant. Nonparametric ANOVA and Dunn's post hoc analyses were used for multiple-group comparisons.

### Ethics statement

Mice were cared for according to the recommendations of the NIH, published in the Guide for the Care and Use of Laboratory Animals. All techniques used were reviewed and approved by the University of North Dakota Institutional Animal Care and Use Committee (IACUC) under the protocol 1503–3.
